# Diameter control of ultrathin zinc oxide nanofibers synthesized by electrospinning

**DOI:** 10.1186/1556-276X-9-267

**Published:** 2014-05-29

**Authors:** Yingjie Liao, Takeshi Fukuda, Norihiko Kamata, Makoto Tokunaga

**Affiliations:** 1 Department of Functional Materials Science, Graduate School of Science and Engineering, Saitama University, 255 Shimo-Okubo, Sakura-ku, Saitama 338-8570, Japan; 2 Comprehensive Analysis Center for Science, Saitama University, 255 Shimo-Okubo, Sakura-ku, Saitama 338-8570, Japan

**Keywords:** Zinc oxide nanofiber, Diameter control, Crystallization, Electrospinning

## Abstract

Electrospinning is a versatile technique, which can be used to generate nanofibers from a rich variety of materials. We investigate the variation of a zinc oxide (ZnO)-polyvinylpyrrolidone (PVP) composite structure in morphology by electrospinning from a series of mixture solutions of ZnO sol–gel and PVP. Calcination conditions for the crystallization of ZnO nanofibers and removal of the PVP component from the ZnO-PVP composite nanofibers were also studied. The progression of the ZnO-PVP composite structure from grains to nanofibers was observed, and ZnO-PVP nanofibers as thin as 29.9 ± 0.8 nm on average were successfully fabricated. The size of the resultant ZnO-PVP composite nanofibers was considerably affected by two parameters: the concentrations of zinc acetate and PVP in the precursor solution. The concentration of zinc acetate particularly influenced the diameter distribution of the ZnO-PVP nanofibers. The ZnO-PVP nanofibers could be subsequently converted into ZnO nanofibers of a pure wurtzite phase via calcination in air at 500°C for 2 h.

## Background

One-dimensional zinc oxide (ZnO) nanostructures have attracted considerable attention within the last decade because of unique characteristics such as large aspect ratio, high electron mobility, and electrical and optical anisotropy [1,2]. Their potential applications in various functional devices, including sensors, solar cells, photodetectors, etc., have been noted [3,4]. Most reported methods for synthesizing one-dimensional ZnO nanostructures follow vapor-solid, vapor-liquid–solid, solution-solid, and solvothermal routes [2,5,6].

Electrospinning is a simple and versatile method along the solution-solid route for producing oxide nanofibers [4,7-10]. Although extensive investigations on the synthesis of ZnO nanofibers by electrospinning, including geometrical directional alignment [11], hydrophobicity [12], electrical properties [3,13], and growth of nanograins [14], have been reported, size control of ZnO nanofibers, especially on the 10-nm scale, has been seldom addressed. Such research, however, is important not only for understanding the mechanism of the electrospinning process but also for widening the field of geometry-dependent applications of ZnO nanofibers.

## Methods

In this work, a mixture of ZnO sol–gel solution and polyvinylpyrrolidone (PVP) (*M*_w_ = 1,300,000, Aldrich, St. Louis, MO, USA) in ethanol was used for electrospinning [15,16]. In a typical procedure, 43.9 mg of Zn(CH_3_COO)_2_ · 2H_2_O was first dissolved in a monoethanolamine (MEA)-2-methoxyethanol solution at room temperature. The molar ratio of MEA to zinc acetate was kept at 1.0, and the concentration of zinc acetate was 0.1 mol/L. The resultant mixture was stirred at 60°C for 30 min to obtain a transparent and homogeneous solution. Then an ethanol solution containing 0.2 g PVP was added to the ZnO sol–gel solution, and the mixture was loaded into a glass capillary with a 100-μm inner diameter at the blunt tip.

Stable high voltage between 0 and 20 kV was generated by a power supply (ETM3-20K01PN1, Element, Sagamihara-shi, Kanagawa, Japan) and applied to the solution through a copper wire in the glass capillary. In addition, an indium tin oxide (ITO)-coated glass substrate (25 mm × 25 mm) was placed perpendicular to the axis of the capillary at a distance of 10 cm from its tip as a counter electrode. This counter electrode was connected to the ground along with the high-voltage power supply.

Three groups of samples were electrospun at 6.0 kV from the precursor solutions, which contained 0.1, 0.4, and 0.75 M zinc acetate, respectively. PVP solution was added into the precursor solution before electrospinning at concentrations varying from 0.02 to 0.14 g/mL for each group. A portion of the synthesized ZnO nanofibers were treated at 300°C in air for 10 min, and the others were calcined at 500°C in a programmable furnace for 2 h. Scanning electron microscope (SEM) images were taken using a field-emission SEM (S-4100, HITACHI, Chiyoda-ku, Japan) operated at an accelerating voltage of 15 kV. The diameters of these fibers were quantitatively evaluated using their high-magnification SEM images. Transmission electron microcopy (TEM) images were taken using a Tecnai G2 20 microscope operated at 200 kV. The X-ray diffraction (XRD) pattern was recorded with a D/MAX Ultima III diffractometer (Cu Kα radiation) at a scanning rate of 0.02°/s in 2*θ* ranging from 20° to 80°.

## Results and discussion

**Figure 1:**
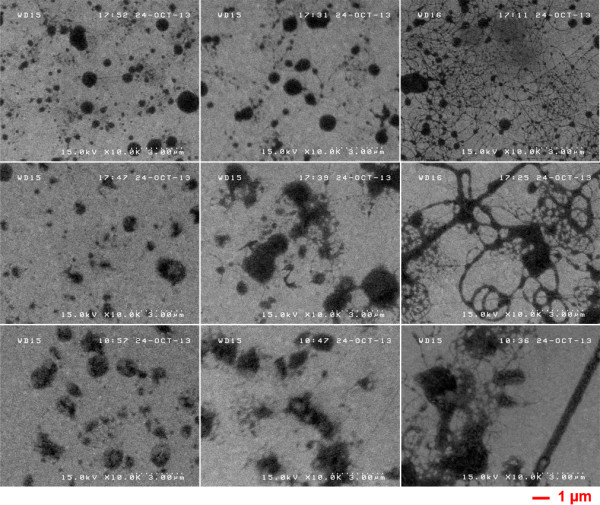
**SEM images of the ZnO-PVP composite structure electrospun from a mixture of ZnO sol–gel and PVP solution.** Concentrations of zinc acetate are 0.1 M (top row), 0.4 M (middle row), and 0.75 M (bottom row); those of the PVP solution are 0.02, 0.04, and 0.06 g/mL from the left to the right column, respectively.

Figure 1 shows SEM images of the ZnO-PVP composite obtained. Progression from grains to nanofibers is observed as the PVP concentration increases from 0.02 to 0.06 g/mL. Comparing the three images in the first row of Figure 1, only ZnO-PVP grains of various sizes are observed in the left image. As the PVP concentration is increased to 0.04 g/mL, a few ZnO-PVP nanofibers appear among ZnO-PVP grains in the middle image. When the PVP concentration is increased to 0.06 g/mL, ZnO-PVP nanofibers become predominant (right image). A similar progression from grains to nanofibers is also seen in the lower two rows (0.4 and 0.75 M zinc acetate) of SEM images in Figure 1. These results indicate that it is not the molar concentration of zinc acetate but the PVP concentration which determines the formation of ZnO-PVP nanofibers.

**Figure 2:**
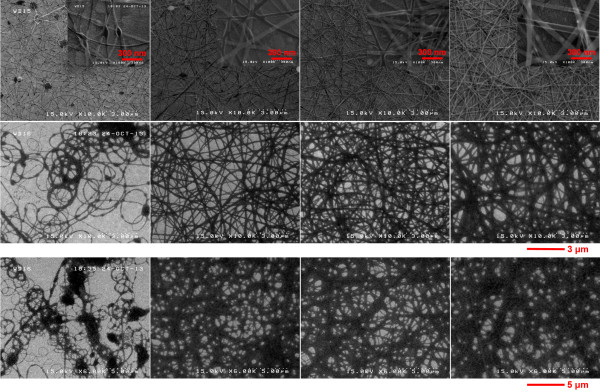
**SEM images of the ZnO-PVP composite nanofibers electrospun from a mixture of ZnO sol–gel and PVP solution.** Concentrations of zinc acetate are 0.1 M (top row), 0.4 M (middle row), and 0.75 M (bottom row); those of the PVP solution are 0.08, 0.12, and 0.14 g/mL from the left to the right column, respectively. High-magnification SEM images (1,100 nm × 900 nm) are shown as insets.

Figure 2 shows the change in the diameter of the ZnO-PVP composite nanofibers when the PVP concentration is adjusted from 0.08 to 0.14 g/mL. Evidently, the diameter of the resultant nanofibers increases steadily with the PVP concentration in all three rows. It is worth noting that the beads present in the top row images (0.1 M zinc acetate) become less prominent with the growth of the nanofibers: this can be attributed to the increase in viscosity of the precursor solution [17]. These results suggest that the concentration of PVP in the precursor solution plays a significant role in determining not only the size of the resultant nanofibers but also the absence of the beads. When comparing the three groups of samples, we find that a precursor solution of relatively high molar concentration of zinc acetate also induces the formation of thicker ZnO-PVP composite nanofibers. Moreover, the nanofibers synthesized with 0.1 M zinc acetate are more uniform than those in the other two groups. In general, the use of zinc acetate and PVP at lower concentration led to the formation of thinner ZnO-PVP composite nanofibers with more beads.

**Figure 3:**
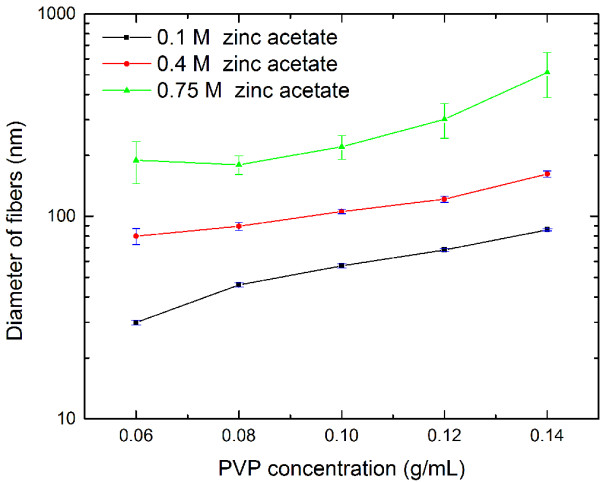
**Statistics for the diameter of the ZnO-PVP composite nanofibers.** The nanofibers were synthesized with the precursor containing 0.1, 0.4, and 0.75 M zinc acetate. Both the mean value and standard error are calculated from 50 measurements.

**Figure 4:**
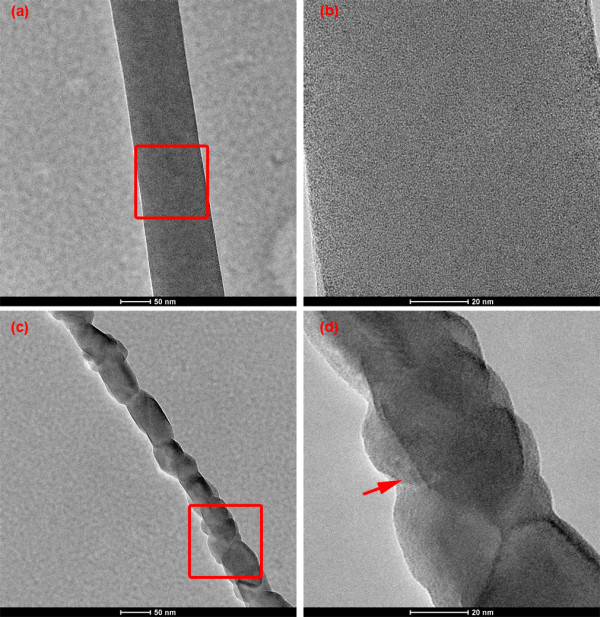
**TEM images of the fibers electrospun from a solution containing 0.1 M zinc acetate and 0.12 g/mL PVP.** After calcination **(a, b)** at 300°C for 10 min and **(c, d)** at 500°C for 2 h.

**Figure 5:**
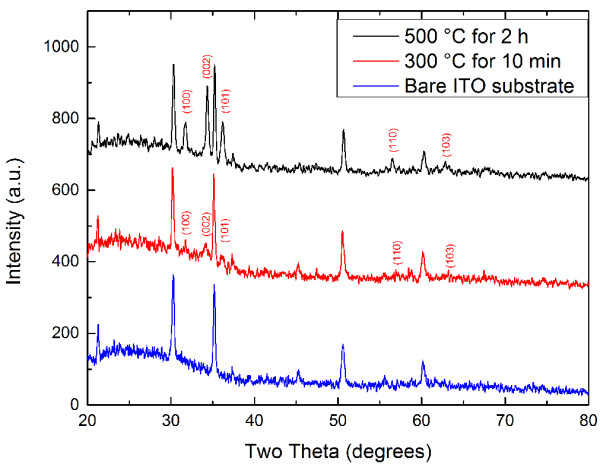
XRD patterns of the fibers calcined at 300°C for 10 min and at 500°C for 2 h.

In order to analyze the effect of the precursor solution on the size of the resultant nanofibers quantitatively, we measured the diameter of the nanofibers from their high-resolution SEM images and plotted the mean of 50 measurements with a corresponding standard error for each sample (Figure 3). For the fibers synthesized with the precursor solution containing 0.1 M zinc acetate, the diameter of the nanofibers increase almost linearly with increasing PVP concentration as shown in Figure 3. The diameter of the finest fibers in this group is 29.9 ± 0.8 nm, which is much smaller than that of any fibers reported in previous papers [8,18]. In the case of 0.4 M zinc acetate, the diameter of fibers increased superlinearly from 79.9 ± 7.1 to 162.0 ± 5.5 nm as the PVP concentration increased from 0.06 to 0.14 g/mL. Comparing the fibers synthesized with given PVP concentration, we found that their diameter increases considerably with the molar concentration of zinc acetate. We also noticed that the standard error of the mean diameter for the fibers synthesized with the precursor solution containing 0.4 and 0.75 M zinc acetate, especially the latter, is larger than that in the case of 0.1 M zinc acetate, which implies that the concentrated ZnO sol–gel solution disturbed the balance of electrospinning set up by the PVP component. In general, an increase in the molar concentration of zinc acetate in the precursor solution not only made the resultant fibers larger in diameter but also contributed to greater nonuniformity in the distribution of the diameter.In order to investigate the microscopic structure of ZnO nanofibers obtained under different calcination conditions, TEM analysis was carried out. The diameter of as-synthesized fibers is around 120 nm before calcination. Figure 4a,b and Figure 4c,d show TEM images of the fibers after being calcined at 300°C for 10 min and again at 500°C for 2 h, respectively. The fibers retained similar shape and diameter after calcination at 300°C for 10 min (see red square in Figure 4a). It is difficult to identify ZnO grains even from the magnified image in Figure 4b, which suggests that the ZnO did not crystallize sufficiently due to the incomplete removal of the PVP in the fibers. The XRD pattern of the ZnO-PVP composite nanofibers also confirmed this point. These results imply that the ZnO-PVP composite nanofibers need a higher calcination temperature and longer calcination duration to remove the PVP content and improve the crystallinity of ZnO. The sample calcined at 500°C for 2 h, on the other hand, is comprised of single isolated ZnO grains (see red square in Figure 4c). The diameter of the fiber shrinks down to about 50 nm. In addition, lattice images are clearly observed in Figure 4d, indicating that each grain is crystalline ZnO. The growth direction of the crystalline ZnO is indicated by a red arrow in Figure 4d. These results reveal that calcination at 300°C for 10 min is insufficient for the crystallization of as-synthesized ZnO-PVP composite nanofibers and grains of crystalline ZnO are formed after calcination at 500°C for 2 h. X-ray diffraction patterns of these fibers also confirm this point. Figure 5 shows the XRD patterns of ZnO-PVP composite nanofibers after calcination at 300°C for 10 min and after calcination at 500°C for 2 h. From the XRD patterns, it is clear that the fibers calcined at 300°C for 10 min are amorphous in nature. Obvious diffraction peaks come from the substrate used for XRD measurement. Characteristic peaks for ZnO are rather weak and obscure, which indicates that only few portions of crystalline ZnO are present under this calcination condition. After calcination at 500°C for 2 h, five diffraction peaks at 31.76°, 34.34°, 36.20°, 56.50°, and 62.84° appear, corresponding to (100), (002), (101), (110), and (103) of the wurtzite crystal structure, respectively. All of the five diffraction peaks are consistent with the reported data for ZnO of a wurtzite hexagonal phase. No characteristic peaks for other impurities, except for the substrate, were found. This means that the phase of the fibers obtained after calcination at 500°C for 2 h is rather pure. These observations imply that the calcination condition plays an important role in removing the PVP component from the composite fibers and improving the crystallinity of ZnO nanofibers.

## Conclusions

In summary, we have demonstrated that the diameter of electrospun ZnO-PVP composite nanofibers can be controlled in the range from hundreds of nanometers down to less than 30 nm. The effects of two key factors, the molar concentration of zinc acetate in the ZnO sol–gel solution and the concentration of PVP in the precursor solution, on the morphology and diameter of the electrospun fibers were discussed, and the calcination condition for generating pure crystalline ZnO nanofibers was also investigated. Pure wurtzite-phase ZnO nanofibers with a clear lattice image in the TEM observation were formed after calcination at 500°C for 2 h. We hope to apply these results to the manufacture of ultrathin ZnO nanofibers for solar cells with increased contacting area and better charge collection efficiency, which is currently underway in our laboratory. We believe that the diameter control method described here may extend the application of ZnO nanofibers to more diameter-dependent devices.

## Abbreviations

ITO: indium tin oxide; MEA: monoethanolamine; PVP: polyvinylpyrrolidone; SEM: scanning electron microscope; TEM: transmission electron microcopy; XRD: X-ray diffraction; ZnO: zinc oxide.

## Competing interests

The authors declare that they have no competing interests.

## Authors’ contributions

YJL fabricated the samples, performed the related characterization, and drafted the manuscript. TF and NK supervised the sample analysis and revised the manuscript. MT carried out the TEM measurement. All authors read and approved the final manuscript.
